# The early career resilience experience of generation Z newly graduated registered nurses in standardized training in the emergency department: a qualitative study in Shanghai

**DOI:** 10.1186/s12912-024-02043-3

**Published:** 2024-06-06

**Authors:** Peng Han, Yingying Sun, Huifeng Chen, Yue Liu, Shuyang Liu, Jing Wang, Chunwei Chi, Zhenjuan Dai, Jing Chen, Li Zeng, Jinxia Jiang

**Affiliations:** 1 Emergency Department, Shanghai Tenth People’s Hospital, School of Medicine, Tongji University, Shanghai, 200072 China; 2 Human Resource Department, Shanghai Tenth People’s Hospital, School of Medicine, Tongji University, Shanghai, 200072 China; 3 Department of medical affairs, Shanghai Stomatological Hospital & School of Stomatology, Fudan University, Shanghai, 200433 China; 4 Emergency department, Song Jiang District Central Hospital, Shanghai, 201699 China; 5 College of Design and Innovation, Tongji University, Shanghai, 200092 China; 6 Nursing Department, Tongji Hospital, School of Medicine, Tongji University, Shanghai, 200065 China

**Keywords:** Resilience, Generation Z, Newly graduated registered nurses, Standardized training, Qualitative study

## Abstract

**Background:**

The period of standardized training is a transitional stage when Generation Z newly graduated registered nurses (Gen Z NGRNs) change their role from student to nurse. Affected by the COVID-19, they lack clinical practice and practicum experience in emergency departments in their university studies. At the beginning of career, they are under great pressure. Resilience is one of the factors that reduce their stress level and increases endurance. It is of interest to understand how this representative group of nurses gained and played the experience of resilience early in their careers.

**Objective:**

To explore Gen Z NGRNs’ experience and process of resilience, to provide a new perspective and theoretical basis for psychological rehabilitation or intervention of medical staff who experienced transition shock.

**Methods:**

This study employed a qualitative design based on the phenomenological approach. 18 nurses from a third-level class-A hospital in Shanghai who participated in standardized training in emergency department were enrolled using purposive sampling. Data collection was through in-depth and semi-structured interviews and continued until reaching data saturation.

**Results:**

The investigation uncovered three themes and ten subthemes. Pressure and challenge contained high workload and high risk coexist, death’s stress response, more emergencies and high professional requirements. Coping and adaptation contained team help, psychological restructuring, peer support, transformational leadership. Reflection and planning contained enhance learning, appreciate life.

**Conclusions:**

Our study described the embodiment and coping experience of the physical and mental stress faced by Gen Z NGRNs during their standardized training in the emergency department. It is emphasized that nurse educators should pay attention to the character and actual needs of Gen Z NGRNs, explore and formulate strategies, so as to guide NGRNs to quickly adapt and grow in the new role. The ultimate goal is to increase nurse retention and improve the quality of nursing.

## Introduction

The department of emergency medicine is the department with the most concentration of severe patients, the most abundant types of diseases, and the most difficult rescue tasks in the hospital system. It is the department that all emergency patients must contact after admission. With the development of medical technology, the emergency department has gradually evolved into an emergency medical service and research center integrating emergency diagnosis, emergency treatment and intensive care, providing the most timely life-saving channel for acute, severe and critical patients [1]. However, the emergency department has the characteristics of more emergencies and critical patients, high mortality of patients, high work intensity and occupational exposure risk, which puts forward strict requirements for the medical staff of the emergency department, who need to have the ability to judge and solve problems independently, and take emergency treatment measures in a limited time to treat critically ill patients. The medical level, the quality of nursing work and the comprehensive quality of medical staff in the emergency department determine the cure rate of patients, and reflect the overall level of the hospital [2]. In China, all nursing students spend about 30 h to complete the specialized course of “Acute and Critical Care Nursing”, and in the clinical practice, they spend 1–2 months in the emergency department, where they develop basic skills in emergency care, which is helpful to their work and improves their theoretical knowledge. Considering the complexity of emergency care, it is necessary to pay close attention to the training and development of new forces in the emergency department, and improve their professional quality through standardized training methods.

**Fig. 1:**
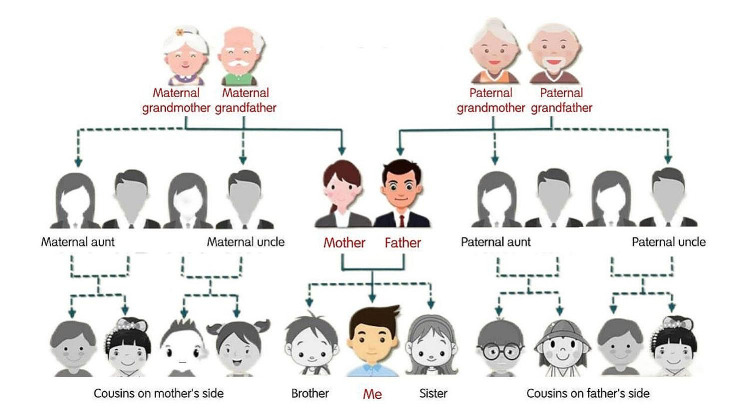
China’s Gen Z “4-2-1” family diagram

Newly graduated registered nurses (NGRNs) are nurses who have obtained the qualification of nursing post after graduation from university and have just entered the hospital nursing post for less than 2 years [3]. Although NGRNs have experienced the learning of professional knowledge in school and the training of clinical practice, they are in the initial stage of entering the formal work in the hospital. The majority of NGRNs in China today come from Generation Z (born between 1995 and 2012), the latest generation of graduates who have completed their nursing education. Generation Z (Gen Z) in China is the “Second-Generation of China One” (descendants of the first only children, who are also only children) and constitute about 19% of the country’s population. They live in China’s “4-2-1 families” (four elderly, two young, and one child), with unique intergenerational characteristics. Figure 1 showed the family composition of Gen Z’s parents and grandparents (they have no siblings or cousins). They were born in an era of material abundance, rapid economic development and rapid advances in information technology. Compared to other generations of young people, Gen Z generally received a high level of education, they have broad vision, rational thinking, value the quality of life, and have a unique set of cognitive standards. And they are familiar with the Internet and are the main force in using social media to communicate.

Standardized training is a transitional stage when Gen Z NGRNs change their role from student to nurse. In 2016, the National Health Commission of China issued the “Training program for Newly Nurses (Trial)”, which put forward clear requirements for the training of NGRNs. The aim is to equip them for clinical nursing positions through a two-year systematic education of theoretical knowledge and clinical skills, in which the period in the emergency department is usually 3–6 months [4]. In the emergency department, a preceptor is assigned to each NGRN. These preceptors are responsible for the guidance, supervision and assessment of NGRNs, helping them to build competencies. The qualification of the emergency preceptor is recognized by the hospital nursing authority, and it is necessary to have the medium-grade professional title or above and pass the annual assessment to obtain the qualification. Preceptors are responsible for the training of acute and critical disease nursing and the teaching of clinical skills, as well as the cultivation of professional values and psychological help for NGRNs. The teaching content of standardized training in the emergency department includes the use of first aid equipment and emergency management, risk assessment and pre-control of critically ill patients, emergency management of batch casualties, first aid operation (CPR, simple breathing apparatus, etc.). Only by fully mastering the skills, reaching a certain workload, and obtaining the approval of the emergency preceptor, can the standardized training task be completed. After the training, the NGRNs evaluate and give feedback to the preceptors, so as to determine the amount of extra reward for the preceptors, which improve their teaching literacy.

However, among the medical staff in the emergency department, Gen Z NGRNs in standardized training are the group with greater pressure crisis. Most of them have never or rarely faced with acute and critical cases, and lack rich professional experience and excellent physical quality [5]. At the same time, affected by the COVID-19, some offline courses in the university have been transformed into online teaching. For the protection of nurse students, the university’s nursing practicum rotation plan has been compressed and the clinical practice in the emergency department has been canceled. Due to the lack of sufficient clinical practice and internship experience in the emergency department, they are under great pressure in the early days of entering such a busy department. They are easily affected by knowledge, responsibility, role and relationship, and have feelings and experiences of confusion, doubt, confusion and unclear positioning. This work adjustment disorder is called Transition Shock [6]. Studies have shown that the turnover rate of NGRNs in the first year of their career can reach 4-54% [7]. The insufficient competency of NGRNs caused by subjective or objective factors will have a great impact on the treatment effect and safety of patients, such as medical errors, nosocomial infection and mortality [7].

Resilience can be interpreted as a mental ability or process of an individual. It implies the ability to bounce back or recover easily when confronted by adversity, trauma, misfortune, or change [8]. In addition, psychological resilience is a process through which a series of individual qualities interact dynamically to enable individuals to quickly recover and successfully cope with great pressure and danger. With the development of positive psychology, resilience has attracted more and more attention as a positive psychological quality for individuals to successfully cope with adversity. For nurses, resilience is one of the factors that reduce their stress level and increases endurance. Studies have found that 35.9% of nurses may experience job burnout after experiencing adversity or traumatic events, and nurses with high levels of resilience have higher job satisfaction and better career development [9]. During the transition process of standardized training, Gen Z NGRNs changed their thinking and behavior in the face of stress to maintain psychological balance, and the resilience experience was the tool to support their growth. It is of interest to understand how this representative group of nurses gained and played the experience of resilience early in their careers.

## Materials and methods

### Study design

The researchers conducted a descriptive phenomenological approach to explore the experiences of resilience of Gen Z NGRNs in standardized training in emergency department in Shanghai. It was conducted through face-to-face semi-structured interviews.

### Ethics approval and consent to participate

Informed consent was obtained from all participants. The Institutional Review Board of Shanghai Tenth People’s Hospital (Shanghai, China) approved the study (Approval No. 23KN25). Each participant read the informed consent carefully and signed an Electronic Consent Statement prior to the start of the interview. Permission was obtained from the participants to arrange one-onone individual interviews. All methods were carried out in accordance with relevant guidelines and regulations in this section.

### Participants

A purposive sampling technique in a third-level class-A hospital (the highest level in the classification of medical institutions in China) in Shanghai was used to select participants who have experience of this phenomenon and can clearly express it. The inclusion criteria were as follows: (1) newly qualified nurses in a third-level class-A hospital in Shanghai; (2) born between 1995 and 2012; (3) undergoing standardized training in the emergency department at least three-month; and (4) willing to participate in this study. An invitation was sent via email to ask for purposive sampling of NQNs to participate in individual interviews.

### Data Collection

Semi-structured, face-to-face interviews were conducted in June 2023. As shown in Table 1, we developed a semi-structured interview guide for data collection. Participants shared their resilience experiences and psychological feelings, which were explored until they fully understood emerging themes. The guide commenced the interview with encouraging questions. If necessary, the interviewer would ask more in-depth and specific follow-up questions. It must be emphasized that the participants had no relationship with the researchers before the study began and were previously unknown to them. In addition, two nurses were selected for a pre-interview prior to data collection to ensure the clarity of the questions and to identify any potential problems. The pre-interviews were treated as tests and were excluded from the analysis. Formal interviews lasted 30 to 60 min each, were audio recorded with permission, and participants’ responses, including nonverbal cues and body language during the interviews, were noted. Interviews were conducted by a group of two researchers and two research assistants. Each interview was transcribed verbatim by researchers and analysed concurrently.

**Table 1:** The interview guideline: open questions

No.	Questions
1	What do you think of the current work on standardized training in emergency department?
2	What difficulties have you encountered in your work?
3	How is your current work affecting you?
4	How do you deal with these difficulties?
5	What are your expectations for the work on standardized training in emergency department?
6	What additional perception and experience did you have?
7	In emergency department, what are the most challenging examples of work for you? Please list 3 of them.
8	In emergency department, what are the most satisfying examples of work for you? Please list 3 of them.

### Data analysis

After the interview, the data were analysed separately and immediately by two researchers with skilled analysis experience. Interview data was analysed using Nvivo 12.0, a computer-assisted qualitative data management software. Colaizzi’s phenomenological seven-step method [10] (Table 2) was used for data analysis to complete the extraction of themes and sub-themes regarding the early career resilience experience of NQNs in emergency department. Any disagreement between researchers was resolved by making decisions through discussion until a consensus was reached. The resulting theme structure was returned to the participants, who were asked over the phone whether their real experience had been captured, and if there was any deviation, researchers woule re-analyzed it from the first step.

**Table 2:** Colaizzi’s seven-step process for qualitative data analysis

No.	Data analysis step
1	All interviews were recorded and transcribed. Each transcript was carefully read several times.
2	Researchers re-read, highlighted, and extracted meaningful statements directly related to the perspectives and experiences of Gen Z NGRNs.
3	Meanings from all significant statements were summarized.
4	Identified and organized the formulated meanings into theme clusters. The researchers compared the theme clusters with the original data several times to determine consistency.
5	Exhaustively described the investigated phenomenon of the resilience experience of Gen Z NGRNs.
6	Recognized similar subthemes, identified the basic structure, and obtained the main themes.
7	Returned to the participants to confirm the findings. The authors discussed their disagreements until a consensus was reached.

### Rigor

Four criteria were applied to ensure research rigor: credibility, dependability, confirmability, and transferability. Credibility was ensured through researcher triangulation, data collection triangulation, and participant validation. Dependability was ensured by providing the data to an experienced external researcher for an independent check. In order to ensure confirmability, an audit trail was used to verify that the authors did not bias the findings. Transferability was ensured by clearly describing the background of the study, the sampling method, and the method of data collection and analysis. This study followed the Consolidated Criteria for Reporting Qualitative Research (COREQ) [11].

## Results

**Fig. 2:**
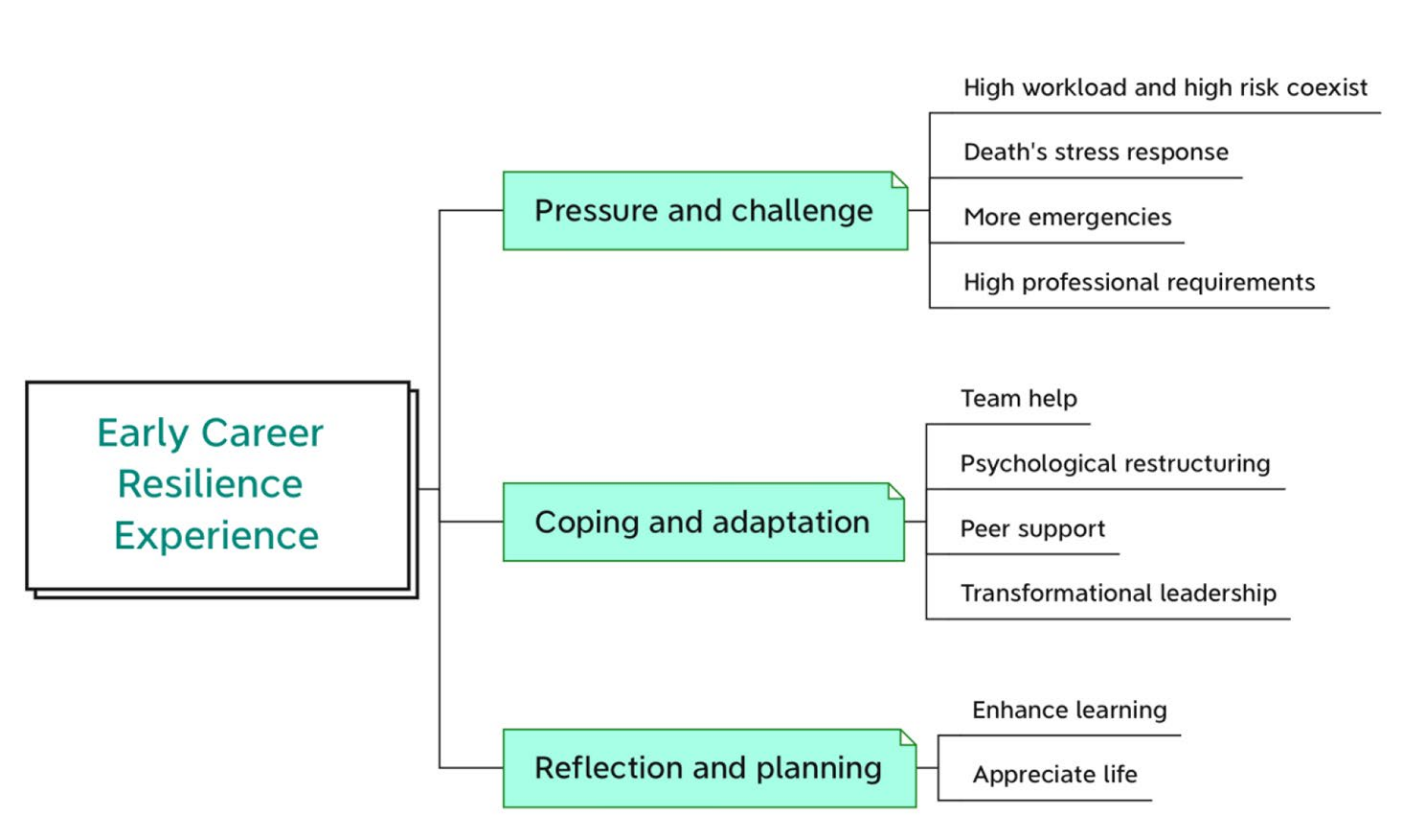
Themes and sub-themes categorized from data

A total of 18 participants (2 males and 16 females) were included in the study. Their average age was 23.1 years. There were 1 participant with diplomas (5.6%) and 17 with bachelor’s degrees (94.4%). The study applied Colaizzi’s method to extract themes to describe the early career resilience experience of Gen Z NGRNs in standardized training in the emergency department. Three themes were emerged: (a) pressure and challenge, (b) coping and adaptation, and (c) reflection and planning. The three themes and ten subthemes are shown in Fig. 2.

### Theme 1: pressure and challenge

#### High workload and high risk coexist

The high work intensity and high risk of the emergency department have never been experienced by the NGRNs of Gen Z. The university clinical practicum of this generation of NGRNs has been affected by the COVID-19 pandemic, and inadequate clinical practicum experience makes them face great work pressure in the early stage of entering the emergency department. Participant 11 described her inability to adapt to emergency department work:

*“Have not seen such critical patients! In the ward rotation, there are stable patients, and the disease is relatively single. I did not go to the emergency department in my internal program and many instruments have not seen, I didn’t get used to it all at once (frown).”*

Participant 10 described the stress caused by her busy work experience:

*“There are many, many treatments every day, sometimes three ambulances come together, all very serious patients, and we may be busy intubating someone, really busy (smile and shake head).”*

Participants 8 considered the writing of patient rating sheets and nursing record sheets to be a high workload in emergency department:

*“Every day to record a variety of patient scores, such as RASS score, APACHE II score, stress injury score, Barthel score, muscle strength score, ha ha, can not say it in one breath… And of course, the nursing notes. I couldn’t stop (smile).*

Due to the critical condition of the patients, the nursing work of emergency department has great risk, for NGRNs, any carelessness may cause adverse consequences. Participants 3 and Participants 16 shared their insights:

*“I am often afraid of making mistakes. Patients have several catheters, I feel I can’t start, and the dosage of some drugs is not easy to adjust the speed.”*

*“I am afraid when I perform gastric lavage operation on the patient, what should I do if perforate?”*

#### Death’s stress response

Gen Z NGRNs are younger and less experienced, and their experience in the emergency department may be their first time facing the death of a patient, which causes a strong stress response that most participants can’t handle. Participant 6 described:

*“At the beginning of the emergency department, I was shocked (wide eyes), how so many critical patients! One day when I saw a 3-year-old child die of suffocation, I was really sad that I could not eat. When I left work, my mind was full of first aid for the child (shake head and puck mouth).”*

Participant 9 described her distress to cope with the death of a peer:

*“There was a man only a few years older than me who committed suicide, and I’m sorry we couldn’t save him, and I keep thinking, what did he go through to get him to this point of no return?”*

#### More emergencies

Emergency nursing work is full of unpredictability. Emergencies happen all the time. Gen Z NGRNs have not yet reached the capacity to be able to cope with these events and therefore feel stressed. For example, patient’s condition suddenly worsens, Participant 4 described:

*“There was a middle shift, met a tetanus patient suddenly tetanus attack, I have never encountered this situation, panic helpless, especially frustrated.”*

Participants felt anxious about patients with serious medical conditions entering the emergency department, Participant 4 and Participant 18 described:

*“There was a sudden batch of car accident patients, with hemopneumothorax, fractures, multiple injuries, it’s too busy.”*

*“I am most afraid of treating patients with sudden acute respiratory failure…”*.

#### High professional requirements

Most of the participants said that the emergency nursing profession has high technical requirements, and the Gen Z nurses who were in the standardized training at the early stage of their career still need to improve their self-requirements in terms of professional ability, and there is a long way to go. Participant 1 described the challenges she faced:

*“Mechanical ventilation patients are very common here, airway management, air bag pressure testing and so on are important knowledge, I have only learned the surface.”*

Learning new skills that had not been touched, Participants 17 described:

*“For the first time I knew what IO (Intraosseous infusion) was, and there was a lot to learn.”*

Participant 5 described the challenges involved in the rescue process:

*“Getting D2W time (the time from arrival at the hospital gate to the first balloon dilation) for STEMI patients is really intense and exciting. In addition to knowing the process of opening emergency green channel, we also have to understand the relevant drug administration, there is too much to learn!”*

### Theme 2: coping and adaptation

#### Team help

Team support is seen as a social resource and plays a role in helping Gen Z NGRNs build and develop resilience. The preceptors in the standardized training played an important role in the development of NGRNs, and all participants felt that they received help from the preceptors. Participant 7 and Participant 4 described:

*“My preceptor is really good, she is very strong, all the rescue is so in one go, feel no extra action, I feel great! She would take the initiative to teach me, such as the principles of ECMO, how to cooperate with the team and so on.”*

The mutual help of team members is effective for improving individual’s organizational commitment and organizational citizenship behavior to the team. Below are description of Participant 11 of how supportive their colleagues were:

*“My colleagues are also very nice, they ask me where I need help, they tell me that nothing is a problem, as long as we work together, there is no problem that can’t be solved!”*

#### Psychological restructuring

A technique psychologists call cognitive restructuring (CR) is one of the most effective ways for people to adapt to pressure, this approach is also known as cognitive reframing or cognitive reappraisal. CR can enable people to turn an experience into a challenge and further turn it into something good for them. Participant 15 shared her experience of restructuring:

*“Take things too hard or blind, in addition to let me go further and further on the road of decadence, can not bring a ray of life to me.”*

Downward comparison principles in social psychology posits that persons experiencing negative affect can enhance their subjective well-being through comparison with a less fortunate other. Such a change of mind can also be seen as a psychological restructuring. Participant 6 described:

*“Seeing so many deaths in the emergency department, I don’t think the difficulties I face are very important.”*

#### Peer support

Participants said that similar experiences with peers made them empathize and feel more connected, peer support had a significant effect on resilience, and the participants described the experience in which they were supported and became confident. Participant 6 described:

*“We talk to each other, found that there is a problem or pressure is not only me, towards the peer, feel that all things are difficult before they are easy, adapt to it.”*

Gen Z is passionate about using online resources and trying new things. Participant 1 and Participant 7 described the way she and her companions relaxed:

*“Our standardized training group members will team up to play esports games, relax! (Laughter)”*.

*“My partner taught me to meditate, to look at my emotions without any critical eyes.”*

#### Transformational leadership

Transformational leadership is an effective style of leadership in health care organizations. Most participants cited transformational leadership from their leaders as an important pillar of their resilience development. Participant 5 described:

*“The head nurse gave me some small tasks, such as authorizing me to prepare knowledge links in the PPT of our department’s nursing rounds. I imported them in the form of video cases and was praised. I felt useful (smile).”*

Psychological contract is an unwritten implicit contract that pays more attention to commitment and reciprocal relationship. With the development of the times and society, Gen Z employees pay more attention to psychological contract. When the organization arouses employees’ sense of happiness, belonging and identity through a solid psychological contract, employees will generate more positive behaviors in return for the organization. Participant 16 was very appreciative of the leadership in the organization, stating:

*“The team atmosphere in the emergency department is very good, and the whole team is full of cohesion. The head nurse asked me about my difficulties and needs from time to time, which made me feel very valued. I hope that I can stay and work here after the standardized training (hands praying).”*

### Theme 3: reflection and planning

#### Enhance learning

Taking an active approach to stress helped the Gen Z NGRNs to develop resilience. All the participants agree that it is very important to strengthen learning and improve their own quality and ability. Participants 13 felt that he had improved himself by learning new skills:

*“I followed the teacher to shoot a popular science video about anaphylactic shock, and I was responsible for the video technology processing. I learned a lot and found my own advantages. Looking back, those so-called difficulties were actually nothing.”*

Participant 6 expressed their desire to improve their abilities through setting plans.

*“I made an action plan, wrote down what I should do at every node of time, and worked hard to improve my professional ability. Only when I was strong, my work efficiency would improve.”*

#### Appreciate life

Resilience emphasizes the growth and rebirth of individuals after trauma or stress. Highly resilient individuals are often able to overcome their emotional icebergs, appreciate life, and move forward from adversity. Participant 18 described her view on adversity at work:

*“The rough past is just a reminder of life. It can teach us how to be a better version of ourselves in the future.”*

Participant 9 described her gratitude for this early work experience:

*“I am very grateful for my current job, although there are many difficulties at the beginning, but it has honed my will and harvested a lot of good things, I am grateful for everything I have now.”*

Gen Z NGRNs are accustomed to using online social platforms to record and share their lives. Here are the descriptions of the Participant 14 and Participant 16:

*“Stay positive, and I will keep track of every little progress, every little success I have on my social media.”*

*“I made a Vlog of standardized training work in the emergency department. The initial difficult time encouraged me to cherish the present and work hard.”*

## Discussion

The study extracted 3 themes to describe the early career resilience experience and process of Gen Z NGRNs in standardized training in the emergency department, provide a new perspective and theoretical basis for psychological rehabilitation or intervention of medical staff who experienced transition shock. Gen Z NGRNs in standardized training faced a great deal of physical and emotional stress at the beginning of their clinical work, which was the norm rather than the exception. These sources of stress come from the lack of professional ability, special job requirements, and work or social environment. Gen Z NGRNs find reasonable ways to cope with stress and achieve self-improvement at the beginning of their careers. They receive training to improve clinical competence, while external support from leaders, teams and peers also plays a vital role. These constitute their early career resilience experience in the emergency department. This study explored the experience and process of Gen Z NGRNs’ resilience, providing a new perspective and theoretical basis for the psychological rehabilitation or intervention of new medical staff also in transition. The following is the specific discussion based on the study findings.

Stress among nursing staff is a global problem, especially among less qualified nurses, who are often identified as a highly stressed group with high turnover rates. A survey of 246 nurses (mostly Gen Z) in standardized training in China showed that the career transition stress (74.28 ± 12.58) and occupational stress (384.56 ± 81.26) of nurses were at a high level [12]. In addition, researchers believe that negative emotions such as anxiety, irritability, and emotional vulnerability are common among NGRNs, which has similarities with our findings [13]. The high stress state reduce the professional experience of NGRNs, such as the huge workload of the emergency department, high exposure risk, death’s stress response, emergencies, etc. The persistence of such a situation poses a threat to patient safety and quality of care, are not conducive to nurse retention, and the social recognition of the profession will also decline, forming a vicious circle. Therefore, in hospital management and education, it is necessary to provide strategies to reduce staff pressure and have a long-term effect.

For Gen Z NGRNs, resilience is activated and manifested under multiple stress stimuli, and is continuously enhanced with the increase of stress level, and finally achieves the target behavior of improving their professional ability and nursing quality. The International Council of Nurses (ICN) further points out that improving resilience can not only promote the physical and mental health of nurses, but also benefit the health and safety of patients and the stable development of health care organizations [14]. Resilience is a continuous, dynamic, multi-dimensional, learned ability, which is the result of internal and external factors. The outcome of resilience after an individual suffers trauma, pressure or challenge depends on the environment, the internal resilience factors and the interaction process between the individual and the environment [15]. Gen Z is a generation with an active mind and good use of resources, whether it is from the Internet social media, or offline learning communication and entertainment activities, they often find a way to suit themselves. So they are more able to motivate themselves and gain resilience experiences to support their growth.

**Fig. 3:**
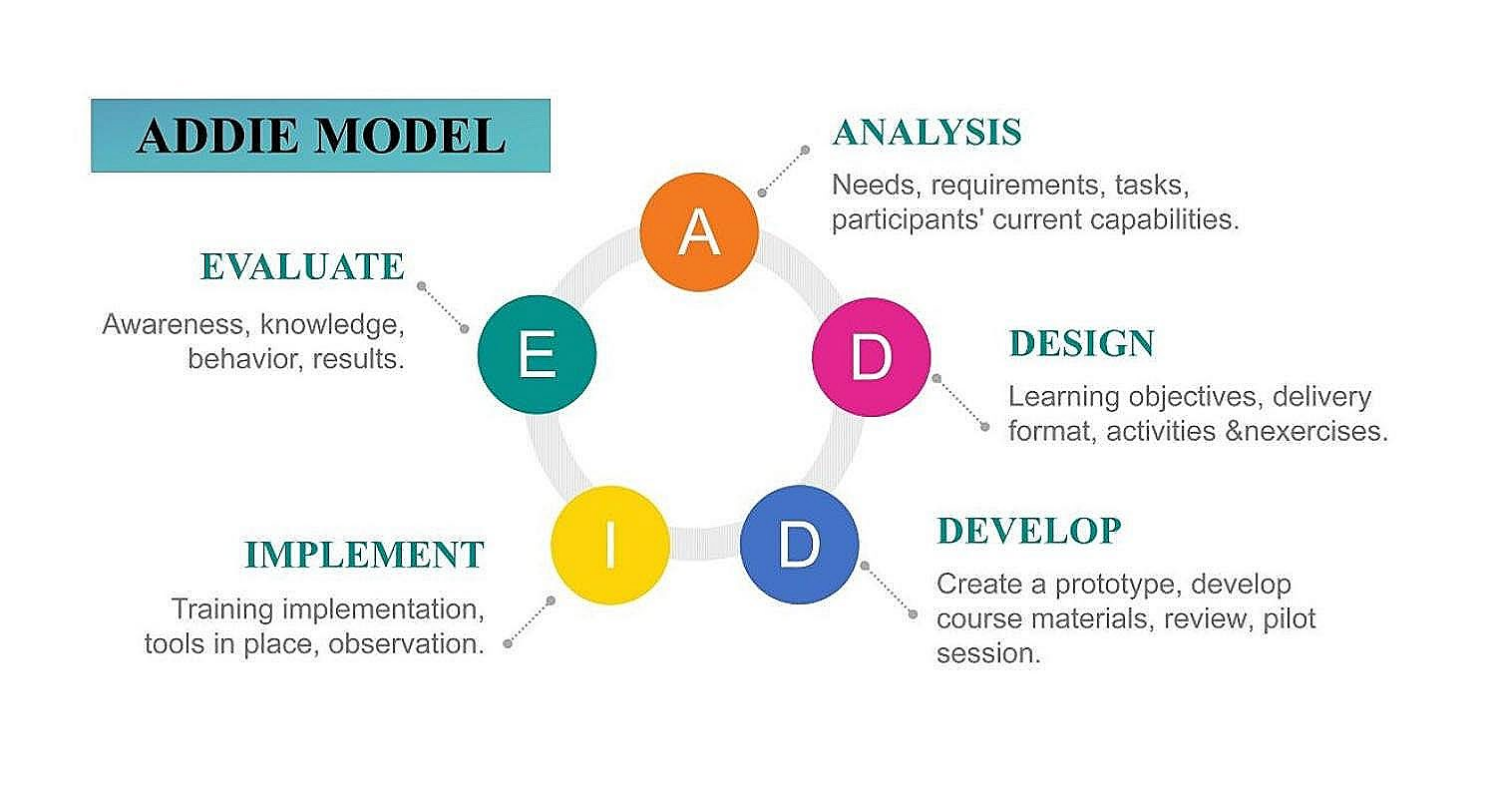
ADDIE training course development model

Team support is seen as a social resource and plays a role in helping Gen Z NGRNs build and develop resilience. As most Gen Z are only children, the academic pressure and lack of companionship in the growing process have limited their contact with the outside world, which has prompted Generation Z to have strong social and emotional needs, constantly searching for a common language to maintain friendship and find their own circle [16]. The gradually formed peer support helps NGRNs integrate into the organization and increase their work commitment and sense of belonging to the organization. In order to alleviate the transition shock of Gen Z NGRNs, putting the individual needs of NGRNs in the first place is a key point of the teaching of standardized training. For example, nursing preceptors should pay more attention to patient safety related training, emergency response ability training, first-aid skills training and other urgently needed contents of nurses to improve NGRNs’ competency. ADDIE model instructional design refers to a systematic approach to developing teaching, including the entire process of Analysis, Design, Development, Implementation, and Evaluation. The method of this system includes several elements: what should be learned? (Setting learning goals); How to learn? (Application of learning strategies); and how to determine that learners have achieved learning outcomes? (Implementation of learning assessment). The curriculum design of standardized training for NGRNs can be based on the ADDIE model (Fig. 3). Nursing educators should analyze existing resources and implement strategies progressively and directionally. Attention should also be paid to the internal understanding and feedback of Gen Z NGRNS on their own roles and practical abilities [16].

Cognitive restructuring is one of the most effective ways for people to adapt to pressure. According to the participants’ description, CR is the re-cognition and transformation of the meaning of negative events in personal, life and work environment in the process of role change under the individual’s subjective will. This is one of the most effective ways to regulate emotions because it enables individuals to take steps to avoid unhelpful emotions or to adjust them after they have already formed, thereby stimulating individual resilience. Gen Z has a distinct personality, expects to achieve self-worth in the work, acquire positive behaviors, and gain the sense of belonging and identity in the work. It is suggested that psychological education should be carried out in the training of NGRNs, and psychological skills should be developed, such as self-suggestion, rumination and downward comparison.

Transformational leadership emphasizes the behavioral style of nursing leaders and the subjective feelings of nurses, and the emotional management of leadership is a favorable means. Studies have shown that resilience levels can be improved by means of emotion management [17]. At the beginning of their careers, Gen Z NGRNs have a strong need to break away from maladaptive situations. Nursing leaders should pay more attention to the psychological state of NGRNs in daily work, take timely measures to remove their bad emotion, and actively communicate with them. Such leadership also includes listening to the demands of employees, encouraging them to express their emotions, dealing with their concerns and opinions in a timely manner, avoiding negative psychological conditions caused by overwork or unfair treatment, and developing reasonable work systems and scientific work processes according to the workload and personnel allocation ratio. Such intervention makes Gen Z NGRNs get great psychological support. The transformational leadership style based on NGRNs’ adaptation to needs helps build NGRNs’ resilience.

With the development of modern nursing discipline, the ability of qualified clinical nurses is increasingly required. Gen Z with independent personality is more proactive in learning. They are good at planning and have clear goals and actions for their pursuit [18]. At the same time, they have much richer network resources, which is different from the past. For example, they are better at using the Internet to gather information, take courses or share communication to decompress. Gen Z has a more optimistic attitude towards life, which is related to their successful life experiences and education. They want to do most things in their own way and at their own pace, choose to be positive, and appreciate life rather than complain, which is a sign of self-confidence. It plays a promoting role in the cultivation of resilience.

Studies have found that there is a significant internal relationship between nurses’ turnover intention and individual resilience [19]. Nursing educators should consider how to implement external strategies to help NGRNs survive the transition shock at the beginning of their careers and improve their resilience, which is related to the construction of nursing teams and the quality of clinical nursing. Various forms of education and ability training are encouraged, for example, by targeting Gen Z NGRNs’ strengths and weaknesses in standardized training, adding mindfulness workshops, death education, peer counseling, role model education, and other methods could be used to train NGRNs to better cope with negative events. Ongoing education and guidance can protect traumatized nurses from absorbing or internalizing unmanageable emotions, which may lead to compassion fatigue. This can help them cope with events more calmly, develop resilience, and boost their careers. Team resilience is a protective factor for individual resilience, manifested as the collective psychological state of team members’ shared cognition, motivation, or emotions [20]. When team adversity is perceived, team members invoke their positive psychological resources and extend individual resilience to the team level through interpersonal interactions [21]. Measures to improve team resilience, including simulation education and team management of clinical aggression training, multimodal resilience training program, stress management and resilience training, and so forth, are effective in promoting individual resilience. Most critically, medical institutions should pay attention to the mental health of medical personnel as a key link, establish a special mental health support organization for medical personnel, and provide long-term and effective support and protection for employees.

## Conclusions

This study described the embodiment and coping experience of the physical and mental stress faced by Gen Z NGRNs during their standardized training in the emergency department. It highlight the role of individuals, organizations and social relationships in the development of resilience. It is emphasized that nurse educators should pay attention to the character and actual needs of Gen Z NGRNs, and explore and formulate strategies from the past experience of standardized training practice, so as to guide NGRNs to quickly adapt and grow in the new role. The ultimate goal is to increase nurse retention and improve the quality of nursing. At the same time, our study provides a valuable basis for the future study of other medical staff’s resilience intervention.

## Data Availability

The original contributions presented in the study are included in the article, further inquiries can be directed to the corresponding authors.
